# *Leccinum molle* (Bon) Bon and *Leccinum vulpinum* Watling: The First Study of Their Nutritional and Antioxidant Potential

**DOI:** 10.3390/molecules21020246

**Published:** 2016-02-20

**Authors:** Filipa S. Reis, Lillian Barros, Anabela Martins, M. Helena Vasconcelos, Patricia Morales, Isabel C. F. R. Ferreira

**Affiliations:** 1 Mountain Research Center (CIMO), School of Agriculture, Polytechnic Institute of Bragança, Apartado 1172, Bragança 5301-855, Portugal; freis@ipatimup.pt (F.S.R.); lillian@ipb.pt (L.B.); amartins@ipb.pt (A.M.); 2 Dpto. Nutrición y Bromatología II, Facultad de Farmacia, Universidad Complutense de Madrid (UCM), Pza Ramón y Cajal, s/n, Madrid E-28040, Spain; patricia.morales@farm.ucm.es; 3 i3S - Instituto de Investigação e Inovação em Saúde da Universidade do Porto, Rua Alfredo Allen, 208, Porto 4200-135, Portugal; hvasconcelos@ipatimup.pt; 4 Cancer Drug Resistance Group, Institute of Molecular Pathology and Immunology of the University of Porto, IPATIMUP, Rua Júlio Amaral de Carvalho, 45, Porto 4200-135, Portugal; 5 Laboratory of Microbiology, Department of Biological Sciences, Faculty of Pharmacy of the University of Porto, Rua de Jorge Viterbo Ferreira 228, Porto 4050-313, Portugal

**Keywords:** chemical profile, nutritional value, nutraceuticals, bioactive compounds, antioxidant potential

## Abstract

This work presents the chemical profile of two edible species of mushrooms from the genus *Leccinum: Leccinum molle* (Bon) Bon and *Leccinum vulpinum* Watling, both harvested on the outskirts of Bragança (Northeastern Portugal). Both species were prepared and characterized regarding their content in nutrients (*i.e.*, free sugars, fatty acids and vitamins), non-nutrients (*i.e.*, phenolic and other organic acids) and antioxidant activity. To the best of our knowledge, no previous studies on the chemical characterization and bioactivity of these species have been undertaken. Accordingly, this study intends to increase the available information concerning edible mushroom species, as well as to highlight another important factor regarding the conservation of the mycological resources—their potential as sources of nutraceutical/pharmaceutical compounds. Overall, both species revealed similar nutrient profiles, with low fat levels, fructose, mannitol and trehalose as the foremost free sugars, and high percentages of mono- and polyunsaturated fatty acids. They also revealed the presence of bioactive compounds, namely phenolic (e.g., gallic acid, protocatechuic acid and *p*-hydroxybenzoic acid) and organic acids (e.g., citric and fumaric acids) and presented antioxidant properties.

## 1. Introduction

Few studies exist on the *Leccinum molle* (Bon) Bon and *Leccinum vulpinum* Watling mushroom species. In the literature it is possible to find a description of the morphological features of *L. molle* [1], and some ecological [2,3,4] and enzymatic [5,6] studies with *L. vulpinum* are available. As far as we know, these are the only studies performed to date, so even considering they are two species of edible mushrooms, they do not seem to be the most consumed by aficionados. However, their interest from the point of view of obtaining nutraceuticals cannot be excluded. Furthermore, apart from trying to valorize these natural resources by providing new information about their potential as a source of bioactive compounds and nutraceuticals, this study also aims to help in the construction of wild edible mushrooms databases.

Since Bragança (Portugal) is one of the regions of Europe with greater mycological biodiversity, it is important to undertake a careful identification and characterization of a large number of wild mushroom species. In addition, being also a region where mushroom picking is a tradition, the scientific community plays an important role in the formation of the general population. Furthermore, public awareness of the need for a careful and healthy diet has increased. In this sense, the consumption of vegetable proteins is increasingly advised and has been increasing over the years. This is due to many reasons including the prevalence of animal diseases, shortage of animal protein worldwide, the strong demand for healthy food, religion, as well as economic reasons [7]. In this regard, mushrooms have been recommended as an alternative protein source to meat, since they are rich in proteins, essential amino acids, vitamins and essential minerals [7,8]. Additionally, as further demonstrated in this work, many researchers have shown that mushrooms are also low in calories and fat [9,10,11,12,13]. Moreover, their primary (e.g., polysaccharides and glycoproteins) and secondary (e.g., phenolic compounds) bioactive compounds, make them a source of nutraceuticals and molecules having medicinal functions such as antioxidant, immunomodulating, and antitumour activity [14,15].

The present study is the first to quantify the nutritional and antioxidant potential of the aforementioned *Leccinum* species, paving the way for new and future studies related to the bioactivity of these species and wild mushrooms in general. Moreover, we intend to valorize these natural resources by advocating species conservation.

## 2. Results

### 2.1. Nutrient Composition

The nutrient composition of the studied species is presented in Table 1.

Regarding fat contents, there were no significant differences between both samples (*p*-value 0.1090). Moreover, both species revealed a low fat content (2.80–2.97 g/100 g dw) compared to proteins (10.48–13.07 g/100 g dw) and particularly carbohydrates (72.94–78.43 g/100 g dw).

From a nutritional standpoint, it is also important to identify the soluble sugars present in the samples. The soluble sugars identified in the studied species were fructose (3.06–4.52 g/100 g dw), mannitol (2.68–11.32 g/100 g dw) and trehalose (2.71–8.31 g/100 g dw) (Table 1).

Considering the fatty acids composition, both species revealed a similar profile, with palmitic acid (C16:0), stearic (C18:0), oleic (C18:1n9) and linoleic acid (C18:2n6) being the main fatty acids present (Table 1). Although palmitic and stearic acids are saturated fatty acids (SFA), these are present in much lower quantities in both species, compared to oleic (a monounsaturated fatty acid—MUFA) and linoleic (a polyunsaturated fatty acid—PUFA) acids. In addition to the lower values, both species revealed similar SFA contents, without significant differences between them (16.94% and 17.09%).

Apart from the abovementioned nutrients, we also assessed the presence of certain vitamins, namely some isoforms of vitamin E (especially known for its antioxidant activity). α- and β-tocopherol were present in both species, but only *L. vulpinum* revealed the presence of the γ-isoform (Table 1). In fact, γ-tocopherol was the prevailing isoform in this species, which contributed heavily to the much higher value of total tocopherols (527.61 µg/100 g dw) compared with *L. molle* (25.36 µg/100 g dw).

### 2.2. Non-Nutrient Composition

The study of some compounds other than nutrients, *i.e.*, phenolic and other organic acids, was performed in both samples. The results are summarized in Table 2.

Analysing the results obtained, oxalic, quinic, citric and fumaric acids were the main organic acids identified. Oxalic and fumaric acid were found in both mushrooms (0.11–1.04 g/100 g dw). However, the profiles differ due to the presence of quinic acid only in *L. vulpinum* (0.13 g/100 g dw) and citric acid in *L. molle* (2.62 g/100 g dw).

**Figure 1:**
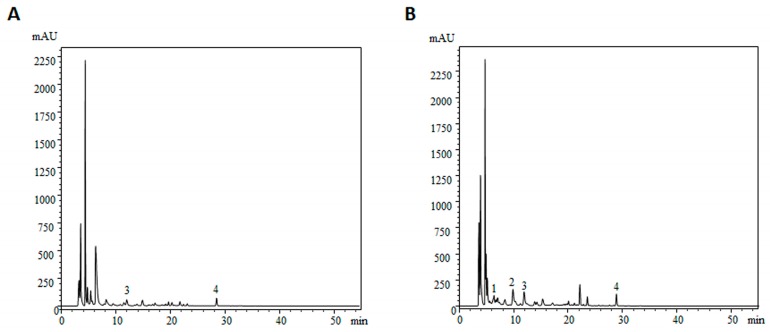
Phenolic acids profile of *L. molle* (**A**) and *L. vulpinum* (**B**) recorded at 280 nm. 1: gallic acid; 2: protocatechuic acid; 3: *p*-hydroxybenzoic acid and 4: cinnamic acid.

Regarding phenolic acids, the profiles also differ substantially among species (Table 1; Figure 1). *L. molle* has only present in its chemical constitution *p*-hydroxybenzoic acid (0.06 mg/100 g dw) and the related compound cinnamic acid (0.13 mg/100 g dw). On the other hand, the *L. vulpinum* profile, besides *p*-hydroxybenzoic acid (0.09 mg/100 g dw) and the related compound cinnamic acid (0.17 mg/100 g dw), also includes gallic acid (0.08 mg/100 g dw) and protocatechuic acid (0.35 mg/100 g dw).

### 2.3. Antioxidant Potential

The antioxidant properties of the samples were evaluated through different assays (Table 3). In order to estimate the reducing power of the samples, two different methodologies were performed: the Folin-Ciocalteu assay and the ferricyanide/Prussian blue assay. To evaluate the radical scavenging capacity, the 2,2-diphenyl-1-picrylhydrazyl (DPPH) radical was used, and to assess the lipid peroxidation inhibition, the β-carotene/linoleate and TBARS assays were performed.

Generally, *L. vulpinum* revealed the highest antioxidant potential. This species displayed the highest reducing power, exhibiting the highest levels of reducing compounds evaluated by the *Folin-Ciocalteu assay* (30.14 mg GAE/g extract). Assessed through the ferricyanide/Prussian blue assay, the highest reducing power also belonged to *L. vulpinum* (lowest EC_50_ value—0.54 mg/mL).

This species also revealed the highest free radical scavenging activity (1.19 mg/mL) and lipid peroxidation inhibition, assessed through the β-carotene bleaching inhibition (0.11 mg/mL) and by the TBARS assay (0.03 mg/mL).

## 3. Discussion

### 3.1. Nutrient Composition

As aforementioned, the nutritional value of the studied species was evaluated in order to recognize and value these species as healthy food. Table 1 shows that both species have low fat content, being richer in proteins and mostly in carbohydrates. Actually, the latter are described as the representative compounds of mushrooms, constituting nearby one-half of mushroom dry matter [11]. However, this group includes several compounds, such as sugars (monosaccharides, their derivatives and oligosaccharides) and the reserve and structural polysaccharides (glycans). So, given that it was intend to evaluate the nutritional value of the mushrooms, the soluble sugars presented by both species was assessed. The soluble sugars identified in the studied species were fructose, mannitol and trehalose (Table 1). Mannitol and trehalose are very common in mushrooms [11]. Regarding fructose, there are some studies relating the presence of this sugar to the trophism of the species. According to some authors [9], this sugar is mainly present in mycorrhizal species, which could justify its identification in these *Leccinum* species. However, this compound has already been identified in saprotrophic species [13]. Therefore, its presence seems then to be related to different factors other than the trophism.

Overall, both species revealed a typical nutritional profile described for mushrooms [10,11], so that they can be referred to as valuable nutritional healthy foods, since they are rich in protein and poor in calories and fat.

Moreover, although the fat levels are low, these include primarily unsaturated fatty acids. As presented in Table 1, although the saturated palmitic and stearic acids represented part of the main fatty acids found in the target species, oleic (MUFA) and linoleic (PUFA) acids were identified in much higher quantities. These findings are important both for the chemical characterization of the species as well as to promote their health benefits. It is well known that many beneficial effects have been attributed to the consumption of unsaturated fatty acids, particularly in relation to prevention of cardiovascular diseases [16,17].

As described in the previous section (Results), vitamin E was also found in the present study. Given the importance of this vitamin in preventing lipid peroxidation [18], its consumption through our daily diet is of the utmost importance. Regarding the obtained results, α- and β-isoforms were present in both species, while γ-tocopherol was only present in *L. vulpinum* (Table 1). This difference can be explained by the specificity of production of different metabolites characteristic of each species. As described in literature [19], some compounds may be produced by all varieties of a particular species or genus during their normal metabolism, while others may be specific of an organism.

According to the findings of this work, and presented in Table 1, both *L. molle* and *L. vulpinum* seem to be valuable healthy foods, being a source of mono- and oligosaccharides, mono- and polyunsaturated fatty acids and vitamin E.

### 3.2. Non-Nutrient Composition

The study of nutrient composition is very important to valorise the studied *Leccinum* species, since it provides information about the importance/benefits of their consumption. However, it is also important to study other molecules that may be biologically active. For this reason, an analysis of phenolic and other organic acids was undertaken in both samples (Table 2).

As mentioned above, both profiles were quite different between species. Therefore, some particular compounds may be mostly produced by a specific species, or their production may be related to the surrounding environment (e.g., possible subjection to stress conditions). In fact, the activation of secondary metabolism and increased production of phenolic compounds in response to various stress conditions is well documented [20].

As previous referred, although organic acids are considered non-nutrients, they are considered important molecules given their biological activities. For example, citric acid is known worldwide for its antioxidant activity and as a natural conservative. This makes *L. molle* as an alternative source of such interesting molecules. Quinic acid is also known for its antioxidant potential [21], which may be an indicator of such activity by *L. vulpinum* species. The other organic acids, oxalic and fumaric acids are also recognised bioactive compounds with pharmacological properties [22,23].

As presented in Table 2 and Figure 1, the chemical profile of *L. vulpinum* is richer in phenolic acids. The presence of these compounds is important because they are pointed out as biologically active molecules. They are often associated with hyperglycaemia and hypertension prevention [24], beneficial cardiovascular effects [25], or the antioxidant, antimicrobial and antitumor potential of the species [26,27]. Although there is a plethora of phenolic compounds occurring in nature, in our study (as in many studies about mushrooms) only the precursor cinnamic acid and two groups of phenolics were identified: some hydroxybenzoic acids (gallic, protocatechuic and *p*-hydroxybenzoic acids) and hydroxycinnamic acids (*p*-coumaric acid). It should be noted that although flavonoids are occasionally reported as compounds present in mushrooms, these matrices do not have the biosynthetic pathway to produce them. Thus, it is believed that the flavonoids exceptionally present in some mushroom species, could be due to the symbiotic relationships established with plants [28]. We also should bear in mind that the chemical composition of mushrooms depends on the environment in which they grow. This is applied even more to secondary metabolites, such as phenolic compounds, which are usually produced in response to stress conditions [29]. Nonetheless, these compounds are present in small amounts in our sample, and the bioactivity present in this study could be correlated with these compounds, but also could be related to other compounds not identified in this study. Thus, the obtained results indicate that *Leccinum* species are a good source of nutrients and nutraceuticals, and their consumption is also a way to obtain phenolic and other organic acids.

### 3.3. Antioxidant Potential

The antioxidant properties of the samples were evaluated through different assays (Table 3). As mentioned, *L. vulpinum* revealed the highest antioxidant potential in all the performed assays. As expected, the bioactive properties of foods are directly related to the bioactive molecules present in their chemical constitution. Therefore, the antioxidant properties of the studied fruiting bodies must be related to some of the compounds studied in this work to present such antioxidant activity. *L. molle* showed higher levels of organic acids (3.95 g/100 g dw), which may be the basis of its antioxidant activity. On the other hand, *L. vulpinum* exhibited greater contents of phenolic acids and much higher values of tocopherols (527.61 µg/100 g dw). Thus, vitamin E and phenolic acids may be largely responsible for the higher antioxidant activity of the species *L. vulpinum*. However, these results do not invalidate that other molecules (e.g., vitamin C, carotenoids, lycopene) may also be responsible for these properties. 

## 4. Experimental Section

### 4.1. Standards and Reagents

Acetonitrile 99.9%, *n*-hexane 95% and ethyl acetate 99.8% were of HPLC grade and obtained from Fisher Scientific (Lisbon, Portugal). The fatty acids methyl ester (FAME) reference standard mixture 37 (standard 47885-U) was purchased from Sigma (St. Louis, MO, USA) as were also other individual fatty acid isomers, standards of sugars, tocopherols, and organic acids, Trolox (6-hydroxy-2,5,7,8-tetramethylchroman-2-carboxylic acid) and phenolic standards. Racemic tocol (50 mg/mL), was purchased from Matreya (Pleasant Gap, PA, USA). 2,2-Diphenyl-1-picrylhydrazyl (DPPH) was obtained from Alfa Aesar (Ward Hill, MA, USA). All other reagents were acquired from specialized sellers. Water was treated in a Milli-Q water purification system (TGI Pure Water Systems, Greenville, SC, USA).

### 4.2. Mushroom Species and Sample Preparation

*Leccinum molle* (Bon) Bon and *Leccinum vulpinum* Watling wild samples were collected in Bragança in the northeastern region of Portugal in the autumn of 2012. Authentications were undertaken at the Polytechnic Institute of Bragança and voucher specimens were deposited at the herbarium of the School of Agriculture of the Polytechnic Institute of Bragança, Portugal. After lyophilisation (FreeZone 4.5 model 7750031, Labconco, Kansas City, MO, USA), all the samples were milled and mixed to obtain a fine dried and homogeneous powder (≈20 mesh), and then stored in a desiccator, until the time of the analysis.

### 4.3. Nutrient Composition

#### 4.3.1. Nutritional Value

The nutritional value of the studied samples was calculated based on protein, fat, carbohydrate and ash contents obtained following standard procedures [30]. The crude protein content (N × 4.38) of the samples was estimated as nitrogen content by the macro-Kjeldahl method; the crude fat by extracting a known weight of milled samples with petroleum ether, by means of a Soxhlet apparatus; and the ash content assessed after incineration at 600 ± 15 °C. Total carbohydrates were calculated by difference. Energy was calculated according to Regulation (EC) No. 1169/2011 of the European Parliament and of the Council, of 25 October 2011, on the Provision of Food Information to Consumers [31], following the equation: Energy (kcal/100g dw) = 4 × (g protein + g carbohydrate) + 9 × (g fat).

#### 4.3.2. Soluble Sugars

Soluble sugars were assessed through chromatographic techniques—high performance liquid chromatograph (HPLC)—following a procedure already described [32]. The HPLC apparatus consisted of a pump (Smartline system 1000, Knauer, Berlin, Germany), a degasser system (Smartline manager 5000) and an auto-sampler (AS-2057 Jasco, Easton, MD, USA), coupled to a refraction index (RI) detector (Knauer Smartline 2300). The chromatographic separation was achieved with a Eurospher 100-5 NH_2_ column (5 µm, 4.6 × 250 mm, Knauer) operating at 30 °C (7971 R Grace oven). The mobile phase was acetonitrile/deionized water, 70:30 (*v/v*) at a flow rate of 1 mL/min. The identification of the sugars was undertaken by comparing their peak relative retention times with commercial standards. Data were analysed through the Clarity 2.4 Software (DataApex, Podohradska, Czech Republic). Finally, the quantification was based on the RI signal response of each standard, relying on the internal standard (IS, raffinose) method and using calibration curves obtained from the commercial standards of each compound. The results were expressed in g per 100 g of dry weight.

#### 4.3.3. Fatty Acids

Before the analysis of fatty acids, a trans-esterification procedure was performed, following a procedure previously reported [32]. The detection was also made by chromatographic techniques, in this specific case, by gas chromatography (GC). The GC equipment was composed by a gas chromatograph (DANI 1000, Contone, Switzerland), equipped with a split/splitless injector and a flame ionization detector (FID). Separation was achieved using a Macherey–Nagel (Düren, Germany) column (50% cyanopropyl-methyl-50% phenylmethylpolysiloxane, 30 m × 0.32 mm i.d. × 0.25 μm df). The oven temperature program was as follows: the initial temperature of the column was 50 °C, held for 2 min, then a 30 °C/min ramp to 125 °C, 5 °C/min ramp to 160 ºC, 20 ºC/ min ramp to 180 °C, 3 °C/min ramp to 200 °C, 20 °C/min ramp to 220 °C and held for 15 min. The carrier gas (hydrogen) flow-rate was 4.0 mL/min (0.61 bar), measured at 50 °C. Split injection (1:40) was carried out at 250 °C. The identification was carried out by comparing the relative retention times of the fatty acid methyl esters (FAME) of the samples with commercial standards. The quantification was made using the Clarity 4.0.1.7 Software (DataApex), being the results expressed as relative percentages of each fatty acid.

#### 4.3.4. Tocopherols

Vitamin E isoforms were also separated by HPLC (above mentioned and described), according to a procedure previously described by other authors [33]. The analysis was performed by coupling with a fluorescence detector (FP-2020; Jasco, Easton, MD, USA), which was programmed for excitation at 290 nm and emission at 330 nm. The chromatographic separation was achieved with a Polyamide II (5 µm, 250 × 4.6 mm) normal-phase column from YMC Waters (YMC America, Inc., Allentown, PA, USA) operating at 30 °C. The mobile phase used was a mixture of n-hexane and ethyl acetate (70:30, *v*/*v*) at a flow rate of 1 mL/min. The peaks detected were compared with commercial standards, being the quantification based on the fluorescence signal, using the internal standard (tocol) method. The results were expressed in μg per 100 g of dry weight.

### 4.4. Non-Nutrient Composition

#### 4.4.1. Organic Acids

Organic acids were detected by Ultra Fast Liquid Chromatography (UFLC). Detection was carried out coupling the chromatograph (Shimadzu 20A series, Shimadzu Corporation, Kyoto, Japan) to a photodiode array detector (PDA), using 215 nm and 245 nm as preferred wavelengths. Separation was achieved on a SphereClone (Phenomenex, Torrance, CA, USA) reverse phase C18 column (5 μm, 250 mm × 4.6 mm i.d.) thermostatted at 35 °C. The elution was performed with sulphuric acid (3.6 mM) using a flow rate of 0.8 mL/min. The organic acids found were quantified comparing the area of their peaks recorded at 215 nm with calibration curves obtained from commercial standards of each compound [34]. The results were expressed in g per 100 g of dry weight.

#### 4.4.2. Phenolic Acids

Phenolic acids analysis was performed using the Shimadzu 20A series ultra-fast liquid chromatograph (UFLC, Shimadzu Corporation, equipment described above) [35]. Separation was achieved using a Waters Spherisorb S3 ODS-2 C_18_ (3 μm, 4.6 mm × 150 mm) column thermostatted at 35 °C. The solvents used were: (A) 0.1% formic acid in water; (B) acetonitrile. The elution gradient established was 10% A to 15% B over 5 min, 15%–25% A in B over 5 min, 25%–35% A in B over 10 min, isocratic 50% B for 10 min, and re-equilibration of the column, using a flow rate of 0.5 mL/min. Double online detection was carried out in the PDA using 280 nm as preferred wavelength and in a mass spectrometer (MS) connected to HPLC system via the DAD cell outlet. The quantification of the phenolic compounds identified was made by comparison of the area of their peaks recorded at 280 nm with calibration curves obtained from the commercial phenolic standards: Gallic acid (y = 224,587x − 129,097; *R*^2^ = 0.9997; LOD 0.19 μg/mL; LOQ 0.63 μg/mL); protocatechuic acid (y = 116,749x − 38733; *R*^2^ = 1; LOD 0.29 μg/mL; LOQ 0.97 μg/mL); *p*-hydroxybenzoic acid (y = 164,204x + 12,917; *R*^2^ = 0.9999; LOD 0.11 μg/mL; LOQ 0.36 μg/mL); cinnamic acid (y = 863,668x − 884,517; *R*^2^ = 0.9998; LOD 0.42 μg/mL; LOQ 1.41 μg/mL). The results were expressed in mg per 100 g of dry weight.

### 4.5. Antioxidant Properties

#### 4.5.1. Extraction Procedure

The lyophilized samples (1 g) were extracted by stirring with 40 mL of methanol at room temperature for 1 h and subsequently filtered through Whatman No. 4 paper. The residue was then extracted with an additional portion of methanol (20 mL) for 1 h. The combined methanolic extracts were evaporated under reduced pressure at 40 °C (R-210 rotary evaporator, Büchi, Flawil, Switzerland) and re-dissolved in methanol (20 mg/mL) and stored at 4 °C for further use.

#### 4.5.2. Antioxidant Activity Evaluation

From the stock solution, successive dilutions were made and submitted to the *in vitro* assays already described [35]. The sample concentrations (mg/mL) providing 50% of antioxidant activity or 0.5 of absorbance (EC_50_) were calculated from the graphs of antioxidant activity percentages (DPPH, β-carotene/linoleate and TBARS assays) or absorbance at 690 nm (ferricyanide/Prussian blue assay) against sample concentrations. Trolox was used as a positive control.

- Reducing power

The reducing power of the samples was measured through the Folin-Ciocalteu assay and the Ferricyanide/Prussian blue assays.

The Folin-Ciocalteu assay allows to quantify the total phenolics and other reducing species present in the samples, relying on their electron transfer capability. The absorbance was measured at 765 nm (Analytikijena 200-2004 spectrophotometer, Jena, Germany) and gallic acid was used to obtain the standard curve. The results were expressed as mg of gallic acid equivalents (GAE) per g of extract.

Regarding the Ferricyanide/Prussian blue assay, the methodology of which is based on the capacity to convert Fe^3+^ into Fe^2+^, the absorbance was measured at 690 nm in a ELX800 Microplate Reader (Bio-Tek Instruments, Inc; Winooski, VT, USA).

- Radical scavenging activity

In the performed assay, the purple chromogen 2,2-diphenyl-1-picrylhydrazyl (DPPH) radical is reduced by antioxidant compounds to the corresponding pale yellow hydrazine. This methodology was accomplished using the microplate reader mentioned above, and the absorption measured at 515 nm. The radical scavenging activity (RSA) was calculated as a percentage of DPPH discoloration using the equation: %RSA = [(ADPPH − AS)/ADPPH] × 100, where AS is the absorbance of the solution containing the sample, and ADPPH, the absorbance of the DPPH solution.

- Lipid peroxidation inhibition

The lipid peroxidation inhibition was evaluated through the inhibition of β-carotene bleaching (or β-carotene/linoleate assay) and the TBARS assay.

In the β-carotene/linoleate assay, the presence of antioxidants in the samples and their capacity to neutralize the linoleate free radicals, avoids β-carotene bleaching, which can be evaluated measuring the absorbance at 470 nm. Therefore, β-carotene bleaching inhibition was calculated using the following formula:

(Absorbance after 2 h of assay/initial absorbance) × 100.

The thiobarbituric acid reactive substances (TBARS) assay is based on the decreasing of TBARS caused by the lipid peroxidation inhibition in porcine (Sus scrofa) brain homogenates due to the presence of antioxidants. This decrease can be measured spectrophotometrically at 532 nm. The inhibition ratio (%) was calculated using the following formula:

Inhibition ratio (%) = [(A − B)/A] × 100%,

where A and B were the absorbance of the control and sample solution, respectively.

### 4.6. Statistical Analysis

For each species, three randomly chosen samples were used and all assays were carried out in triplicate. Results were expressed as mean values and standard deviation (SD). The results of each parameter were compared by means of a Student’s t test to determine the significant difference among samples, with α = 0.05. This analysis was carried out using JMP Statistical Discovery V. 10 program (IBM SPSS software, Armonk, NY, USA).

## 5. Conclusions

This work is the first to report the nutritional, chemical and bioactive properties of *Leccinum molle* (Bon) Bon and *Leccinum vulpinum* Watling. Given the chemical composition of both species, we conclude that they are a good food option for inclusion in today’s diet, being a source of essential nutrients, as well as nutraceuticals and other biologically active compounds. They revealed a good nutritional value (low in fat) and proved to be rich in MUFA and PUFA, soluble sugars (other than sucrose or glucose) and vitamin E. Additionally, they also exhibited antioxidant properties, which corroborates the statement of these mushrooms as health foods.

## Figures and Tables

**Table 1:** Nutrients composition of wild samples of *Leccinum molle* (Bon) Bon and *Leccinum vulpinum* Watling (mean ± SD).

Nutrients	*Leccinum molle* (Bon) Bon	*Leccinum vulpinum* Watling	*p-*Value
Ash (g/100 g dw)	5.70 ± 0.55	13.61 ± 0.86	<0.01
Proteins (g/100 g dw)	13.07 ± 1.00	10.48 ± 0.50	0.0156
Fat (g/100 g dw)	2.80 ± 0.16	2.97 ± 0.13	0.1090
Carbohydrates (g/100 g dw)	78.43 ± 0.86	72.94 ± 0.51	0.0007
Energy (kcal/100 g dw)	391.18 ± 0.99	360.41 ± 2.89	<0.001
Fructose (g/100 g dw)	3.06 ± 0.03	4.52 ± 0.14	<0.0001
Mannitol (g/100 g dw)	11.32 ± 0.30	2.68 ± 0.01	<0.0001
Trehalose (g/100 g dw)	2.71 ± 0.09	8.31 ± 0.01	<0.0001
Total sugars (g/100 g dw)	17.09 ± 0.24	15.51 ± 0.14	0.0002
C16:0 (relative percentage)	11.23 ± 0.21	12.25 ± 0.25	0.0011
C18:0 (relative percentage)	1.79 ± 0.01	2.77 ± 00.04	<0.0001
C18:1n9 (relative percentage)	38.61 ± 0.33	27.06 ± 0.09	<0.0001
C18:2n6 (relative percentage)	43.49 ± 0.10	53.60 ± 0.37	<0.0001
SFA (relative percentage)	16.94 ± 0.41	17.09 ± 0.31	0.4377
MUFA (relative percentage)	39.29 ± 0.31	28.59 ± 0.04	<0.0001
PUFA (relative percentage)	43.77 ± 0.10	54.32 ± 0.27	<0.0001
α-Tocopherol (µg/100 g dw)	12.48 ± 0.70	14.76 ± 1.13	0.0137
β-Tocopherol (µg/100 g dw)	12.88 ± 0.99	216.18 ± 0.90	<0.0001
γ-Tocopherol (µg/100 g dw)	nd	296.67 ± 0.19	0.0032
Total tocopherols (µg/100 g dw)	25.36 ± 0.29	527.61 ± 2.22	0.0008

dw: dry weight; nd: not detected; main fatty acids: C16:0 (palmitic acid), C18:0 (stearic acid), C18:1n9 (oleic acid) and C18:2n6 (linoleic acid); 20 more fatty acids were identified in trace amounts; SFA: saturated fatty acids; MUFA: monounsaturated fatty acids; PUFA: polyunsaturated fatty acids.

**Table 2:** Non-nutrient composition of wild samples of *Leccinum molle* (Bon) Bon and *Leccinum vulpinum* Watling (mean ± SD).

Compounds	*Leccinum molle* (Bon) Bon	*Leccinum vulpinum* Watling	*p*-Value
Oxalic acid (g/100 g dw)	0.29 ± 0.03	0.11 ± 0.01	0.0002
Quinic acid (g/100 g dw)	nd	0.13 ± 0.01	<0.0001
Citric acid (g/100 g dw)	2.62 ± 0.16	nd	<0.0001
Fumaric acid (g/100 g dw)	1.04 ± 0.02	0.25 ± 0.01	<0.0001
Total organic acids (g/100 g dw)	3.95 ± 0.19	0.49 ± 0.01	<0.0001
Gallic acid (mg/100 g dw)	nd	0.08 ± 0.01	<0.0001
Protocatechuic acid (mg/100 g dw)	nd	0.35 ± 0.05	<0.0001
*p*-Hydroxybenzoic acid (mg/100 g dw)	0.06 ± 0.01	0.09 ± 0.01	0.0002
Total phenolic acids (mg/100 g dw)	0.06 ± 0.01	0.52 ± 0.05	<0.0001
Cinnamic acid (mg/100 g dw)	0.13 ± 0.01	0.17 ± 0.01	<0.0001

nd: not detected; dw: dry weight.

**Table 3:** Antioxidant properties of the methanolic extracts of wild samples of *Leccinum molle* (Bon) Bon and *Leccinum vulpinum* Watling (mean ± SD).

Activity	Assay	*Leccinum molle* (Bon) Bon	*Leccinum vulpinum* Watling	*p*-Value
Reducing Power	Folin-Ciocalteu assay (mg GAE/g extract)	17.76 ± 0.26	30.14 ± 0.70	<0.001
	Ferricyanide/Prussian blue assay (EC_50_; mg/mL)	2.13 ± 0.01	0.54 ± 0.01	<0.001
Radical scavenging activity	DPPH radical-scavenging activity assay (EC_50_; mg/mL)	10.68 ± 0.55	1.19 ± 0.02	<0.001
Lipid peroxidation inhibition	β-carotene/linoleate assay (EC_50_; mg/mL)	2.23 ± 0.05	0.11 ± 0.01	<0.001
	TBARS assay (EC_50_; mg/mL)	1.48 ± 0.02	0.03 ± 0.00	<0.001

Regarding the *Folin-Ciocalteu* assay, higher values mean higher reducing power; regarding the other assays, the results are presented as EC_50_ values, which mean that higher values correspond to lower reducing power or antioxidant potential. EC_50_ is the extract concentration corresponding to 50% of antioxidant activity or 0.5 of absorbance for the Ferricyanide/Prussian blue assay. Trolox EC_50_ values: 41 µg/mL (reducing power), 42 µg/mL (DPPH scavenging activity), 18 µg/mL (β-carotene bleaching inhibition) and 23 µg/mL (TBARS inhibition). GAE: gallic acid equivalents.
